# The use of collimator angle optimization and jaw tracking for VMAT‐based single‐isocenter multiple‐target stereotactic radiosurgery for up to six targets in the Varian Eclipse treatment planning system

**DOI:** 10.1002/acm2.13360

**Published:** 2021-07-19

**Authors:** Lauren M. M. Pudsey, Dean Cutajar, Alex Wallace, Anastasia Saba, Laurel Schmidt, Andrej Bece, Catherine Clark, Yoshiya Yamada, Giordano Biasi, Anatoly Rosenfeld, Joel Poder

**Affiliations:** ^1^ Centre for Medical Radiation Physics University of Wollongong Wollongong NSW Australia; ^2^ St George Hospital Cancer Care Centre Kogarah NSW Australia; ^3^ Department of Radiation Oncology Memorial Sloan Kettering Cancer Center New York NY USA

**Keywords:** collimator angle optimization, island blocking, jaw tracking, multiple brain metastases, single‐isocenter stereotactic radiosurgery

## Abstract

**Purpose:**

Island blocking occurs in single‐isocenter multiple‐target (SIMT) stereotactic radiotherapy (SRS) whenever targets share multi‐leaf collimator (MLC) leaf pairs. This study investigated the effect on plan quality and delivery, of reducing island blocking through collimator angle optimization (CAO). In addition, the effect of jaw tracking in this context was also investigated.

**Methods:**

For CAO, an algorithm was created that selects the collimator angle resulting in the lowest level of island blocking, for each beam in any given plan. Then, four volume‐modulated arc therapy (VMAT) SIMT SRS plans each were generated for 10 retrospective patients: two CAO plans, with and without jaw tracking, and two plans with manually selected collimator angles, with and without jaw tracking. Plans were then assessed and compared using typical quality assurance procedures.

**Results:**

There were no substantial differences between plans with and without CAO. Jaw tracking produced statistically significant reduction in low‐dose level parameters; healthy brain V10% and mean dose were reduced by 9.66% and 15.58%, respectively. However, quantitative values (108 cc for V10% and 0.35 Gy for mean dose) were relatively small in relation to clinical relevance. Though there were no statistically significant changes in plan deliverability, there was a notable trend of plans with jaw tracking having lower gamma analysis pass rates.

**Conclusion:**

These findings suggest that CAO has limited benefit in VMAT SIMT SRS of 2–6 targets when using a low‐dose penalty to the healthy brain during plan optimization in Eclipse. As clinical benefits of jaw tracking were found to be minimal and plan deliverability was potentially reduced, a cautious approach would be to exclude jaw tracking in SIMT SRS plans.

## 1 INTRODUCTION

The occurrence of brain metastases can be devastating for a patient, greatly impacting quality of life, and without treatment the median survival time from diagnosis is between 1 and 3 months.^1^, ^2^, ^3^, ^4^ The incidence of cancer patients developing one or more brain metastases is estimated at between 20% and 50%^1^, ^2^, ^3^, ^5^, ^6^, ^7^ and this number is rising due to increased surveillance and improved local and systemic treatment resulting in prolonged survival and better extra cranial disease control.^2^, ^3^, ^5^, ^6^ The presence of multiple metastases at once is common; a study by Fabi et al.^2^ of 290 patients treated for brain metastases reported that 41% of them had three or more metastases.

Currently, medical linear accelerator (LINAC)‐based SRS is most often delivered using multi‐leaf collimators (MLCs) via volumetric‐modulated arc therapy (VMAT) and/or dynamic conformal arc therapy (DCAT).^8^ Relative to previously used cone collimators, the shielding provided by the MLC, however, is not optimal due to interleaf leakage or transmission which contributes to the low dose to normal tissue.^9^, ^10^, ^11^

Delivery of VMAT plans is conventionally performed with the LINAC secondary collimator jaws remaining static at the maximum aperture that the MLC achieves during treatment.^10^, ^12^ It is proposed that the interleaf leakage or transmission through the MLC, and hence the low‐dose delivered to normal tissue, may be reduced through the implementation of jaw tracking.^13^ This is a recently developed method involving the secondary collimator jaws moving dynamically throughout treatment to match the MLC apertures as closely as possible, therefore, providing additional shielding.^10^, ^11^ There have been many studies that confirm the improvement in low‐dose parameters via the addition of jaw tracking to VMAT treatment plans, though very few of these focus on treatment of brain cancer or the use of SRS.^9^, ^10^, ^11^, ^12^, ^14^, ^15^ These studies found all plans with jaw tracking to be deliverable,^10^, ^12^, ^15^ however, a study by Wu et al.^15^ reported some variation in quality assurance (QA) pass rates for VMAT plans with jaw tracking compared to fixed jaws while still remaining clinically acceptable. Though current literature in general agrees that jaw tracking results in improved low dose to normal tissue,^9^, ^10^, ^11^, ^12^, ^14^, ^15^ the limited investigations into deliverability of SRS plans implies that further research is required.

**FIGURE 1:**
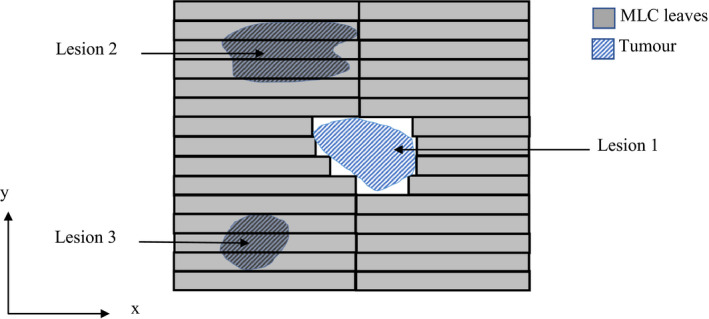
A beam's eye view schematic diagram of an MLC with an aperture shaped to allow a single tumor volume (Lesion 1) to be irradiated while shielding two others (Lesion 2 and Lesion 3). Note, figure is not to scale

Treatment of multiple brain metastases using LINAC‐based SRS is traditionally performed with an isocenter for each lesion centered at the target volume and several arcs for each target.^7^, ^16^, ^17^ Throughout the treatment, only one tumor is irradiated while all others are shielded by the MLC as normal tissue would be (Figure 1). One of the primary concerns with this method is the long treatment time for multiple lesions.^7^, ^8^ Duration of treatment for each isocenter, usually involving several arcs, is between 15 and 20 min^7^, ^16^ meaning treatment times can extend to well over an hour for multiple lesions, requiring the patient to be immobilized for the entire duration. Treatment time is a significant concern not only for patient comfort but also treatment scheduling with available machines.^7^ An alternative method is to treat all targets at once using a single isocenter.

**FIGURE 2:**
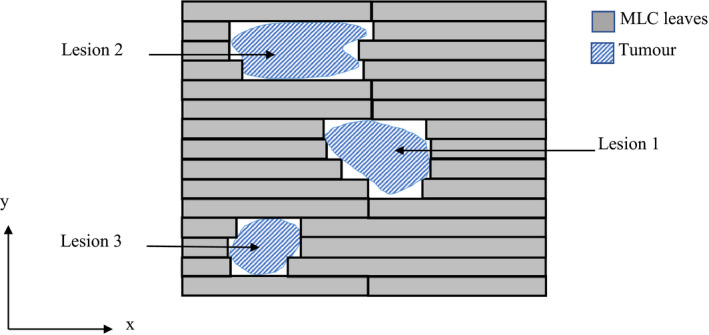
Schematic diagram of an MLC with an aperture shaped to allow all three tumor volumes (Lesion 1, Lesion 2, and Lesion 3) to be irradiated simultaneously. Note, figure is not to scale

Single‐isocenter multiple‐target (SIMT) SRS using VMAT holds the potential to drastically reduce the treatment times for multiple metastases while maintaining the treatment quality.^8^, ^16^, ^17^, ^18^ However, some concern remains regarding low dose to healthy tissue.^16^, ^19^ In SIMT SRS, placement of the isocenter is usually at the geometric center of the target distribution.^16^, ^17^, ^20^ The MLC aperture is shaped so that all lesions are irradiated while healthy tissue is shielded (Figure 2). Some studies have reported a higher level of low‐dose spillage to healthy tissue or increased risk of necrosis when targets are close to each other.^8^, ^16^ The correlation with proximity lends support to the currently leading theory that the dose spillage is mainly due to the issue that has been termed by Kang et al.^19^ as island blocking. Other possible contributions to the higher low dose to normal brain tissue include larger jaw openings and, therefore, increased leakage between leaves.^17^, ^20^

**FIGURE 3:**
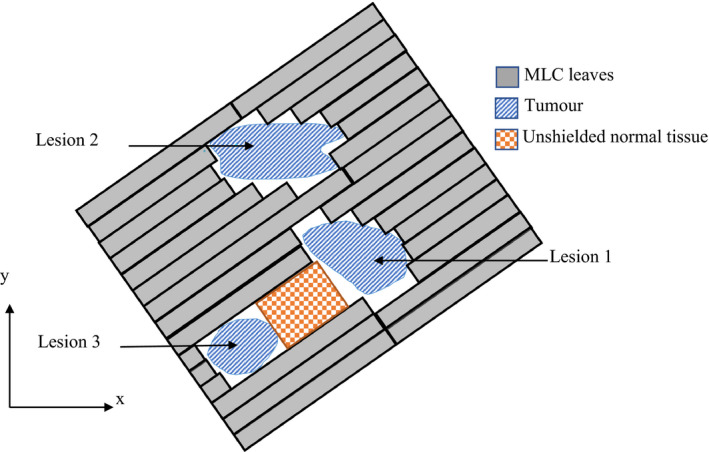
Schematic diagram of an MLC with an aperture shaped to allow all three tumor volumes (Lesion 1, Lesion 2, and Lesion 3) to be irradiated simultaneously but with an area of unshielded normal tissue. Note, figure is not to scale

Island blocking, also referred to as a high‐dose bridge,^7^ occurs when two or more target volumes share one or more MLC leaf pairs.^19^ This results in an area of healthy tissue which is unable to be shielded (Figure 3). Kang et al.^19^ proposed that island blocking could be avoided/reduced by optimizing the collimator angle and improving the plan quality. This optimization involves using an algorithm to select the collimator angle with the lowest level of island blocking.^19^ Since then, several additional studies have utilized varying methods of collimator angle optimization (CAO) to investigate the effect upon treatment quality, reaching a consensus that CAO improves/reduces dose to healthy tissue across all methods of optimization applied.^7^, ^17^, ^20^, ^21^, ^22^ A study by Yuan et al.,^20^ of 10 patients with between 3 and 11 brain metastases, investigated the impact of several factors upon low‐dose spillage including island blocking, jaw tracking, and low‐dose optimization priority in planning. Though jaw tracking and CAO both improved/reduced low‐dose spillage, it was found that the largest impact upon low‐dose spillage was through implementation of a healthy brain optimization objective.^20^

**FIGURE 4:**
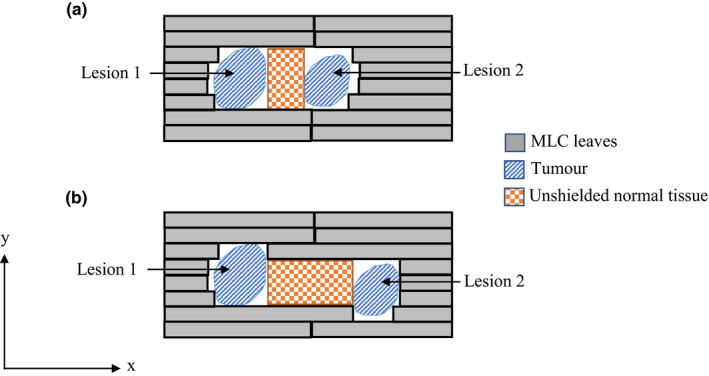
Schematic diagrams of two scenarios with an MLC aperture shaped to allow both tumor volumes (Lesion 1 and Lesion 2) to be irradiated simultaneously. It can be seen that the island blocking area in (a) is much less than that in (b) despite a greater number of MLC leaves being blocked

Several studies have investigated CAO using an algorithm contained in their treatment planning system (Eclipse, version 13.7, Varian Medical Systems).^7^, ^22^ There is little information available on the process involved in this optimization algorithm. Similarly with Phongprapun et al.,^21^ the method used to optimize collimator angles is described only as a dynamic conformal arc technique and any discussion of the processes involved in this is absent. Kang et al.^19^ provides a detailed method of the creation of an algorithm that calculates the optimized collimator angles. However, this proposed method only takes into consideration the overlap in the beam's eye view (BEV) *y*‐direction, therefore, inaccuracies due to variation in distance between tumors are present. This inaccuracy is illustrated in Figure 4, where the tumors in scenario (a) have a larger overlap in the BEV *y*‐direction and more MLC leaf pairs shared than in scenario (b), however, the island blocking area is smaller. Studies by Yuan et al.^20^ and Wu et al.^17^ take this issue into account via different approaches. Yuan et al.^20^ generated MLC aperture patterns for each angle combination and calculated the island blocking area to be the open area of the MLC minus the target area. Wu et al.^17^ offered a solution using projections onto the BEV plane rather than BEV *y*‐axis, however, gave no detailed explanation into the process involved. Despite the consensus among literature of the benefits of CAO,^7^, ^17^, ^19^, ^20^, ^21^, ^22^ currently, access to CAO tools is very limited.

This study aimed to produce a tool that minimizes island blocking through CAO. Additionally, there is limited information available regarding the impact of the existing technique known as jaw tracking upon SIMT SRS. This study aimed to evaluate the effect of jaw tracking across all aspects of treatment quality. Finally, the impact of both techniques upon plan deliverability is assessed.

## 2 MATERIALS AND METHODS

An investigation into the effects of CAO and jaw tracking upon SIMT SRS treatment of multiple brain metastases was undertaken. There were three main methodological processes involved: development of a CAO algorithm; generation of treatment plans and comparison of plan quality parameters; and QA of plans.

### 2.1 Development of a collimator angle optimization algorithm

The CAO algorithm was coded using MATLAB (v.2018a, The MathWorks, Inc.). The collimator angle (*c*) that minimizes the level of island blocking occurring is calculated for the couch angles (*t*) identified by the departmental treatment guidelines and gantry angles (g). These couch angles in degrees are: *t* = 0°, 45°, 90°, 270°, and 315°. The user selects the required couch angles and number of targets in the treatment plan. The area of island blocking that occurs is calculated for every gantry angle (*g*) and summed over the gantry arc creating a total for every combination of specified couch and collimator angles.

The planning target volumes (PTVs) are approximated within the collimator optimization code as six coordinates representing the maximum and minimum displacement in the *x*‐axis, *y*‐axis, and *z*‐axis directions. These are obtained using the following information provided by the user: a center position; diameters in the *x*‐, *y*‐, and *z*‐axis directions; and the isocenter position. In order to replicate treatment, three transformation matrices are required that rotate the PTV coordinates, respectively, to the beam's eye view (BEV) of the collimator: one for gantry motion (equation 1), one for couch angle (equation 2), and one for collimator rotation (equation 3).

(1) $$M_{\mathrm{Gantry}}\left(g\right)=\left(\begin{array}{ccc} \text{cos}\left(g\right) & ‐\text{sin}\left(g\right) & 0\\ \text{sin}\left(g\right) & \cos\theta g & 0\\ 0 & 0 & 1 \end{array}\right)$$

(2) $$M_{\mathrm{Couch}}\left(t\right)=\left(\begin{array}{ccc} \text{cos}\left(t\right) & 0 & \text{sin}\left(t\right)\\ 0 & 1 & 0\\ ‐\text{sin}\left(t\right) & 0 & \text{cos}\left(t\right) \end{array}\right)$$

(3) $$M_{\mathrm{collimator}}\left(c\right)=\left(\begin{array}{ccc} \text{cos}\left(t\right) & 0 & ‐\text{sin}\left(t\right)\\ 0 & 1 & 0\\ \text{sin}\left(t\right) & 0 & \text{cos}\left(t\right) \end{array}\right)$$

The order of multiplication is important as the matrix multiplication does not commute and is as follows:

$$\text{New coordinate vector}\;=\left(M_{\mathrm{collimator}}\left(M_{\mathrm{Gantry}}\cdot M_{\mathrm{couch}}\right)\right)\cdot \text{Original coordinate vector}.$$

**FIGURE 5:**
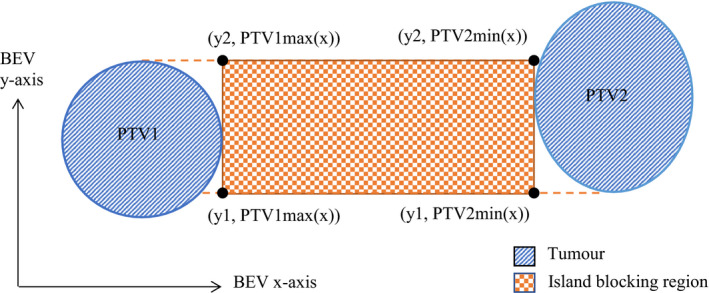
Definition of the boundaries of the island blocking area. In this case, PTV2 has a greater BEV x‐displacement than PTV1; therefore, the minimum x‐coordinate of PTV2 and the maximum x‐coordinate of PTV1 are used

For each gantry angle, the regions of overlap in the BEV *y*‐axis direction must be found. If overlap is present, separation in the BEV *x*‐direction for a pair of PTVs is found and a rectangular region of island blocking is then created as shown in Figure 5. The bounds of the island blocking area are defined using *y*1 and *y*2 which refer to the coordinates of BEV *y*‐axis projection overlap. Depending on which PTV has the greatest *x* displacement, either the maximum or minimum *x* position for each PTV is used for boundary coordinates.

Multiple island blocking regions are accounted for by finding the union of all the island blocking areas created. So that tumors present in the island blocking region are not counted as unshielded healthy tissue, the area of intersection of each PTV and the island blocking region is subtracted from the total island blocking area. The above process is iterated over the gantry arc for each collimator angle and the collimator angle with the least amount of island blocking occurring is selected for treatment.

### 2.2 Generation of treatment plans and plan quality comparison

Treatment plans were generated for 10 retrospective patients with between 2 and 6 brain metastases treated at St George Hospital Cancer Care Centre between 2018 and 2020. The physical attributes and dose prescriptions for each patient are in Table 1. The treatment planning system Eclipse (version 15.6, Varian Medical Systems, Palo Alto, CA) was used to create a total of four plans for each patient: (a) manually selected collimator angle and jaw tracking turned off; (b) CAO and jaw tracking turned off; (c) manually selected collimator angle and jaw tracking turned on; and (d) CAO and jaw tracking turned on. The manually selected collimator angles were those used in the actual treatment of the patients, with collimator angles selected by an expert planner to reduce island blocking based upon the BEV of targets throughout treatment arcs. A different planner used this information and generated all plans used in this study. The isocenter was placed at the geometric center of the targets; for plans with jaw tracking turned off, the secondary collimator jaws were set at the maximum limits of the MLC throughout each gantry arc. A 6 MV flattening filter free beam (6FFF) was used. All plans were made to conform to departmental planning and treatment guidelines.

**TABLE 1:** Physical PTV attributes and dose prescriptions of each patient

	Number of PTVs	Average 3D PTV off‐axis distance (cm)	Range of 3D PTV off‐axis distance (cm)	Average PTV volume (cc)	Range of PTV volumes (cc)	Prescribed dose (Gy)	Number of fractions
Patient 1	3	3.30	2.76–5.19	10.98	5.42–19.50	30	5
Patient 2	3	5.70	5.57–9.01	4.23	0.90–6.40	27	3
Patient 3	4	5.65	5.08–6.40	2.38	0.63–5.34	27	3
Patient 4	6	6.10	5.09–7.94	9.17	1.17–19.85	30	5
Patient 5	3	2.20	1.28–3.01	1.35	0.31–3.11	18	1
Patient 6	3	5.60	4.10–6.73	8.25	2.14–12.96	30	5
Patient 7	2	3.70	3.52–3.80	10.52	7.63–13.42	30	5
Patient 8	2	3.80	3.64–3.96	0.81	0.51–1.11	20	1
Patient 9	5	4.19	3.10–5.69	5.40	0.63–9.73	27	3
Patient 10	2	4.87	4.20–5.59	22.55	3.94–41.96	30	5

**FIGURE 6:**
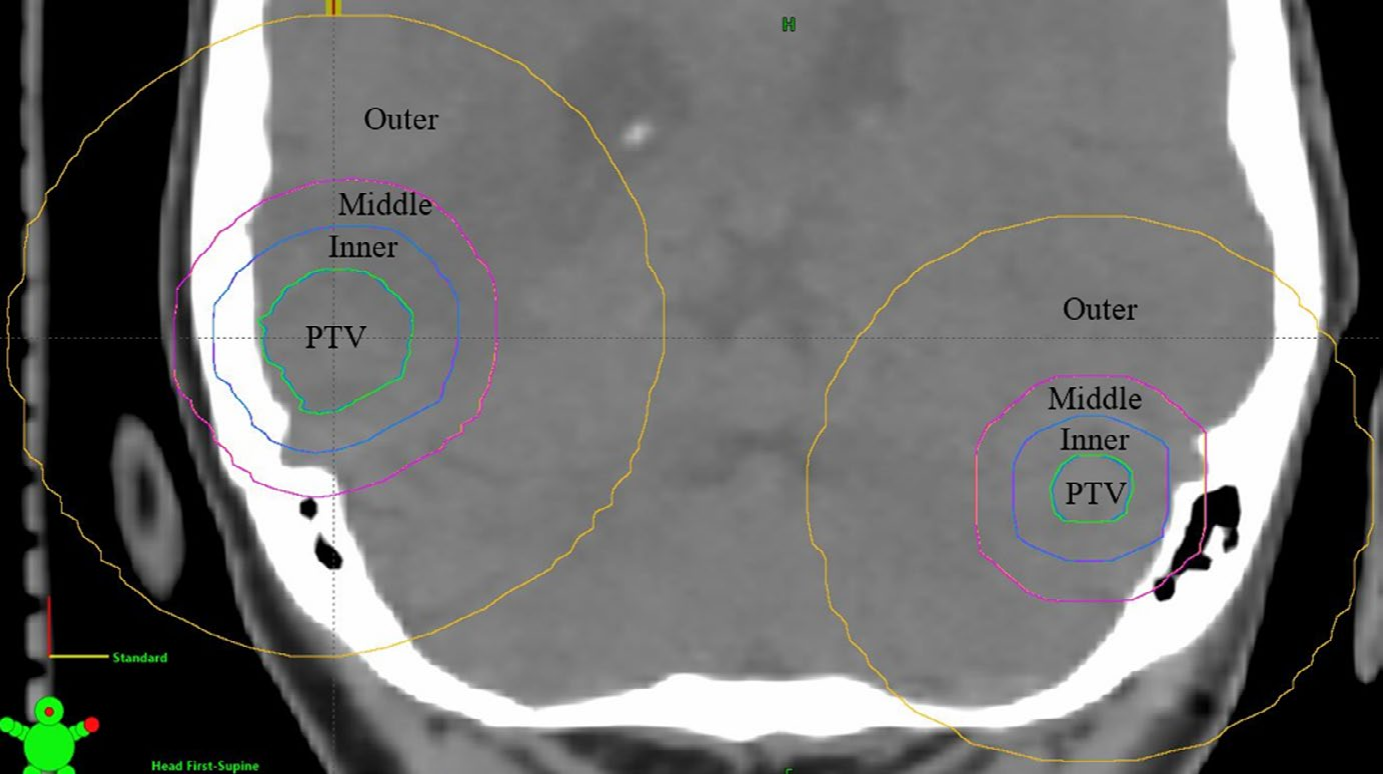
Transverse cross‐sectional CT image of a patient head showing ring‐shaped control regions utilized in the optimization process

Optimization was conducted using several ring‐shaped control regions (Figure 6). The “inner control region” is the volume outside each of the PTVs and inside a boundary which is 0.5 cm outside the PTV margins. The “middle control region” is the volume outside the inner control region and inside a boundary 1 cm outside the PTV margins. The “outer control region” is a volume outside the middle control region and inside a boundary 3 cm outside the PTV margins. An additional normal brain control region is created which is the brain volume minus each of the PTV volumes referred to as “brain‐PTV.” The optimization objectives used, based on the work by Clark et al.,^23^ are in Table 2.

**TABLE 2:** Optimization objective functions used in treatment planning

Control region	Limit type	Volume (%)	Dose (% of prescription)	Priority
PTV	Upper	0	140 (intact) 120 (cavity)	50
	Lower	100	100	125
Inner	Upper	0	98	50
Middle	Upper	0	50	50
Outer	Upper	0	40	50
Brain‐PTV	Upper	1	17	125

The quality of plans was assessed using several parameters: modulation factor (MF) which is defined as the monitor units (MUs) of the plan divided by prescribed dose per fraction in cGy; target coverage (V100%), healthy brain V12 Gy (the volume of healthy brain receiving a dose of 12 Gy) in cc; healthy brain V10% (the volume of healthy brain receiving 10% of the prescribed dose) in cc; healthy brain V25% (the volume of healthy brain receiving 25% of the prescribed dose) in cc; healthy brain mean dose in Gy; conformity index (CI) (the volume of the 100% isodose line divided by the PTV volume); and gradient index (GI) (the volume of the 50% isodose line divided by the volume of the 100% isodose line). Statistical analysis was undertaken using R (v.4.0.2, R core team, 2020) and R studio (v.1.3.959, R studio Team, 2020, PBC) to determine if there was statistically significant difference between these parameters. This was performed via a two‐way ANOVA test comparing parameters across patients and plan types. Therefore, patient data were treated as paired across plan types. If statistically significant difference was found across plan types, a post hoc Tukey's HSD test was undertaken to determine between which plan types this occurred.

### 2.3 Quality assurance

Each plan was copied onto and delivered to a phantom using a Varian TrueBeam (v2.7) LINAC after which gamma analysis^24^ was carried out for each arc. Two sets of dose measurements were taken per plan, as per department protocol, using the SRS MapCHECK device (v1179, Sun Nuclear Corporation, Melbourne, Florida, USA) to intercept as many high‐dose regions as possible (a minimum of 2 PTVs were sampled per plan). This is a device consisting of two parallel slightly offset planar arrays with a total of 1013 N‐type diodes across an active area of 7.7 × 7.7 cm^2^ ^25^ Gamma analysis was undertaken using SNC Patient software (v8.3, Sun Nuclear Corporation) with a threshold of 10%, a distance to agreement of 1 mm, and percentage dose differences of 5%, 4%, 3%, and 2%. Results were averaged across the two measurements. Statistical analysis was undertaken as above.

## 3 RESULTS

### 3.1 Plan quality parameters

Shown in Table 3 are the average values and standard deviations of each plan quality parameter across all 10 patients for each plan type. Variation was observed across plan types. The most notable differences include a reduction in average values of healthy brain V10% when jaw tracking was applied, with the largest difference being 108 cc between the plans with manually selected collimator angles with and without jaw tracking. There was a reduction in average healthy brain V25% when jaw tracking was applied with the largest difference being 19.8 cc between the CAO plan type without jaw tracking and the manual plan type with jaw tracking. Values for both performance indicators, GI and CI, were at clinically acceptable values (CI <1.5 and GI <5) for all individual PTVs across plan types with the exception of extremely small targets (<1.2 cc) which can be expected. Therefore, minor variations among plans were not clinically significant. All other parameters had little to no discernible difference.

**TABLE 3:** Average values and standard deviations of plan quality parameters for each plan type

	Manual angle, no jaw tracking	Optimized angle, no jaw tracking	Manual angle, with jaw tracking	Optimized angle, with jaw tracking
MF	5.17 ± 0.69	5.30 ± 0.65	4.86 ± 0.88	5.29 ± 0.48
Healthy brain V10% (cc)	718.64 ± 302.22	696.84 ± 285.62	610.68 ± 279.21	625.48 ± 266.84
Healthy brain V25% (cc)	159.31 ± 112.39	164.84 ± 111.07	145.04 ± 101.35	151.86 ± 100.33
Plan CI	1.17 ± 0.10	1.21 ± 0.17	1.18 ± 0.11	1.20 ± 0.15
Plan GI	3.11 ± 0.41	3.29 ± 0.70	3.10 ± 0.47	3.23 ± 0.79
Average max PTV dose (%)	142.98 ± 6.25	145.29 ± 8.40	143.40 ± 8.49	145.33 ± 8.65
Average PTV V100% (%)	99.8 ± 0.5	99.8 ± 0.5	99.8 ± 0.5	99.8 ± 0.5

In order to more easily distinguish these quantitatively small variations, the percentage difference between the plan type with neither optimization or jaw tracking and each of the remaining three plan types was found. These average percentage differences in quality parameters are summarized in Table 4. The addition of CAO slightly increased MF while jaw tracking alone reduced MF. For the intermediate dose‐level parameters, the addition of collimator optimization alone increased values, with an 8.77% increase in V12 Gy and a 9.74% increase in V25%. For the lower dose‐level parameters, V10% and mean dose, CAO alone showed little effect. The use of jaw tracking saw a −11.36% change for V25%, −15.58% for V10%, and −9.66% for mean dose but little difference for V12 Gy. The largest reduction in mean dose was between the manual plans with and without jaw tracking and corresponded to a quantitative average value of 0.36 Gy. All values for mean dose were <7.07 Gy across plan types and patients with an average overall of 3.95 Gy. The plan CI worsened slightly by 3.11% when CAO was applied but jaw tracking had no effect. The plan GI also worsened slightly by 5.05% when CAO was applied but again jaw tracking had no effect. For all parameters, the use of both jaw tracking and CAO had similar effect to the combination of the effects of both techniques used alone for example, for V25%, CAO alone showed an increase and jaw tracking alone showed a decrease of similar magnitude and the combination of both techniques resulted in no notable difference. This trend is consistent across all parameters.

**TABLE 4:** Average percentage difference of plan quality parameters when compared with the plan type without collimator angle optimization or jaw tracking

	Optimized angle, no jaw tracking (%)	Manual angle, with jaw tracking (%)	Optimized angle, with jaw tracking (%)
MF	3.71 ± 13.97	−4.97 ± 16.41	3.28 ± 10.38
Healthy brain V12 Gy	8.77 ± 21.34	−0.15 ± 4.42	5.15 ± 20.09
Healthy brain V10%	−0.48 ± 10.08	−15.58 ± 10.12	−9.41 ± 16.28
Healthy brain V25%	9.74 ± 21.60	−11.36 ± 10.76	1.46 ± 20.08
Healthy brain mean dose	0.13 ± 3.99	−9.66 ± 6.29	−6.13 ± 6.44
Plan CI	3.21 ± 7.04	0.74 ± 1.61	2.14 ± 5.20
Plan GI	5.05 ± 9.52	−0.38 ± 2.44	2.90 ± 11.62

A two‐way ANOVA test was undertaken to determine if there was statistically significant difference between plan quality parameters across plan types, a *p* value of 0.05 or greater indicates a null hypothesis. The two‐way ANOVA test indicated significant differences for healthy brain V10% (*p* =< 0.001), healthy brain V25% (*p* = <0.01), and healthy brain mean dose (*p* = <0.001). A post hoc Tukey's HSD test was carried out for each of these three parameters to determine between which plan types the significant difference existed. The results of the post hoc Tukey's HSD test indicated that for healthy brain V10% and healthy brain mean dose, the statistically significant difference was for plans that contained jaw tracking, while for healthy brain V25% the significantly different plan was the one that contained both CAO and jaw tracking.

### 3.2 Quality assurance

**FIGURE 7:**
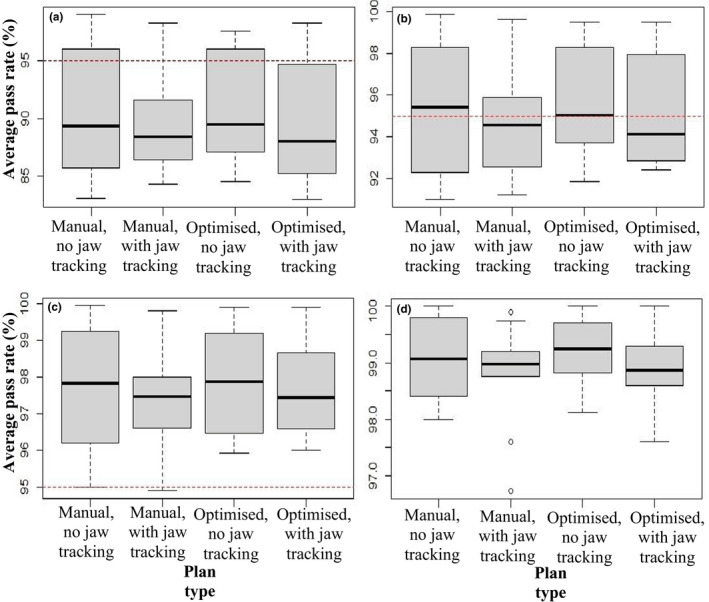
Box and whisker plots of the average pass rate of plans averaged across all patients for the following percentages and distances to agreement: (a) 2%/1 mm, (b) 3%/1 mm, (c) 4%/1 mm, and (d) 5%/1 mm. The dashed red line indicated an acceptable average pass rate of 95%, the black lines are mean values, the boxes are interquartile ranges, the whiskers show maximum and minimum values, and the circles are outliers

The average gamma analysis pass rates for each plan type are shown in Table 5. For the 2%/1 mm criteria, the two plan types that include jaw tracking show a slight decrease in average pass rate of approximately 1% as well as a slight reduction in minimum pass rate of approximately 2%. For the criteria of 3%/1 mm, once again there is a slight decrease in average pass rate when jaw tracking is applied of approximately 0.5% and minimum pass rate of approximately 1.5%. However, these slight variations were not found to be statistically significant. There is very little difference across plan types for the remaining sets of criteria and variables. Though there were no statistically significant differences found, it can be seen from the box and whisker plots in Figure 7 that the average pass rates decrease slightly for plans with jaw tracking included at all four sets of gamma analysis criteria.

**TABLE 5:** Average gamma analysis results across all patients

%/ distance to agreeance	Pass rate (% ± std. dev.)	Manual angle, no jaw tracking	Optimized angle, no jaw tracking	Manual angle, with jaw tracking	Optimized angle, with jaw tracking	F(3,9) value	*p* value
2%/1 mm	Average	90.5 ± 6.1	90.9 ± 4.8	89.8 ± 5.0	89.6 ± 5.4	0.143	0.933
	Min.	82.4 ± 10.0	81.6 ± 9.2	79.6 ± 10.4	79.1 ± 10.9	0.250	0.861
	Max.	96.7 ± 3.4	96.9 ± 3.1	95.9 ± 2.8	96.4 ± 3.1	0.181	0.909
3%/1 mm	Average	95.4 ± 3.4	95.6 ± 2.8	94.9 ± 2.8	95.1 ± 2.6	0.137	0.937
	Min.	89.5 ± 8.4	89.5 ± 6.3	88.2 ± 7.0	88.0 ± 7.4	0.142	0.934
	Max.	98.7 ± 1.5	99.0 ± 1.3	98.4 ± 1.4	98.9 ± 1.0	0.312	0.816
4%/1 mm	Average	97.7 ± 1.7	97.9 ± 1.4	97.5 ± 1.5	97.9 ± 1.3	0.150	0.929
	Min.	93.8 ± 5.3	94.2 ± 4.2	93.0 ± 4.2	94.2 ± 4.2	0.302	0.824
	Max.	99.6 ± 0.7	99.6 ± 0.6	99.6 ± 0.4	99.6 ± 0.5	0.007	0.999
5%/1 mm	Average	99.1 ± 0.7	99.2 ± 0.6	98.8 ± 1.0	98.8 ± 0.7	0.858	0.427
	Min	96.8 ± 3.1	97.3 ± 2.3	95.9 ± 3.2	96.0 ± 2.8	0.514	0.675
	Max.	100.0 ± 0.0	99.9 ± 0.2	100.0 ± 0.1	99.9 ± 0.2	0.793	0.506

## 4 DISCUSSION

### 4.1 Plan quality parameters

Of the intermediate dose‐level parameters, V12 Gy and V25%, the addition of CAO alone increased values, with an 8.77% increase to the mean value of V12 Gy and a 9.74% increase in V25%. The use of CAO alone had very little impact upon the low‐dose level parameters, V10% and healthy brain mean dose, with no notable differences observed. This is contradictory to findings from previous studies which showed a reduction in low‐dose levels when using CAO.^7^, ^17^, ^19^, ^20^, ^21^, ^22^ The variation in results from the expected values are likely due to the optimization objectives used in the treatment planning process. The work by Yuan et al.^20^ found that CAO had very little effect upon dose‐level parameters in comparison to the use of a low‐dose objective in optimization which was employed in this research. The improvement obtained with CAO may be insignificant in comparison to the improvement already in place due to the low‐dose spillage optimization objective. Additionally, Ohira et al.^22^ observed that the treatment planning system at times used the MLC or secondary jaws to completely shield one PTV to avoid island blocking.^22^ This effect was also observed in plans from this study, hence CAO may have become obsolete due to compensations by the treatment planning system.

The use of jaw tracking alone showed very little difference for V12 Gy, however, the lower dose‐level parameters did vary. V25% saw a change of −11.36%, V10% changed by −15.58%, and −9.66% for mean dose. Unlike with CAO, these results are consistent with expectations. Jaw tracking aims to reduce dose leakage and transmission through the MLC^10^, ^11^, ^13^ which for a 120 leaf MLC (Varian Medical Systems) is approximately between 1.6% and 2.5% for X‐ray beams with energy between 6 MV and 18 MV.^11^ This contributes to low‐dose levels without having a large impact upon intermediate dose levels. These results are consistent with findings from previous studies of the impact of jaw tracking upon treatment at various locations in the body.^9^, ^10^, ^11^, ^12^, ^13^, ^14^, ^15^

The results of the ANOVA tests showed that there was highly statistically significant difference across plan types for both V10% (*p* = <0.001) and healthy brain mean dose (*p* = <0.001). The Tukey's HSD post hoc test indicated that this is as a result of jaw tracking alone with a significant reduction when applying jaw tracking to both V10% and healthy brain mean dose. Though, it should be noted that even though the average change in V10% when applying jaw tracking is −15.58%, this corresponds to a quantitative value of just 108 cc and an average change in mean dose of −9.66% is quantitatively −0.35 Gy. From a clinical perspective, these variations are quite small and likely would not play a major role in treatment efficacy.

Statistically significant difference was found for V25% (*p* = <0.01) with a post hoc Tukey's HSD test showing that the only difference was between the plans with optimization but no jaw tracking and plans with jaw tracking but no optimization (*p* = <0.01). Statistical significance for this one combination of plan types but not the two combinations testing the addition of jaw tracking or the two combinations testing the addition of CAO suggests that the difference in V25% has been incorrectly identified as being significant. The accuracy of statistical estimations and testing can be greatly impacted by the presence of extreme results or outliers.^26^ The results contain several outliers which in most cases (including V25%) are in relation to patient data with an average PTV volume of only 0.81 cc. A large percentage difference is actually a very small quantitative variation, creating misleading results.

There is a strong correlation between plan modulation and MUs required for treatment.^27^, ^28^ This study used MF to compare modulation across plans with the addition of CAO resulting in an increase of MF by 3.71% and jaw tracking a change of −4.97%. Despite small variations, there was found to be no statistically significant difference in MF across plan types. Though indicators such as MUs alone are often used to assess plan complexity, this can be technique dependent and additional factors need to be considered to determine an accurate impact upon plan complexity.^28^

Previous studies of SIMT VMAT plans have reported a correlation between lower plan quality and patient geometries with targets in close proximity.^8^, ^16^ Hardcastle and Tome^8^ compared SIMT VMAT and multi‐isocenter conformal arc technique plans finding that the greatest increase to symptomatic radiation necrosis occurred when targets were close together. This would suggest that the greatest improvements due to CAO should be observed in patient plans with low 3D off‐axis distance between targets. In this study, patient 5 had the lowest 3D PTV off‐axis distance of 2.20 cm and possessed better than average % difference (meaning a lesser increase or greater reduction) when CAO alone was applied for V12 Gy, V10%, V25%, CI, and GI. However, patient 6, also with three targets but an off‐axis distance of 5.60 cm, had a better than average % difference in all performance indicators suggesting CAO had a greater effect upon patient 6 than patient 5. This does not support the expectation of more improvement for close proximity targets. Investigating patient 1 with the second smallest average target separation of 3.30 cm, better than average % difference is found for all performance indicators. Patient 3 with an off‐axis distance of 5.65 cm had better than average % difference when CAO was applied for MF, V12 Gy, V25%, and GI. This second comparison between patient 1 and patient 3 suggests a greater effect of CAO for patient 1 with closer target proximity, however, this is in contrast to the first comparison.

Though the relationship between target proximity and impact of CAO is inconsistent and therefore does not allow any trends to be ascertained, there does appear to be a consistent correlation between target size and CAO effectiveness. The two previously discussed target geometries with the more successful application of CAO, belonging to patient 6 and patient 1, have average PTV volumes of 8.94 cc and 10.98 cc, respectively. These are much larger than the average PTV volumes for patient 5 and patient 3 of 1.35 cc and 2.38 cc, respectively, which suggests a greater impact of CAO upon larger targets regardless of target proximity. This trend continues with the largest average PTV volume in this study of 22.55 cc belonging to patient 10 with better than average % difference in all performance indicators. The smallest average PTV volume of 0.81 cc was possessed by patient 8 who also had two PTVs and showed worse than average % difference for all performance indicators. Patient 10 and patient eight had relatively similar average PTV off‐axis differences of 4.87 cm and 3.80 cm once again suggesting that there is no notable correlation between CAO effect and target proximity in this study. Due to the few number of patients in this study that possessed greater than three targets, it is difficult to assess whether number of PTV’s impacted the effectiveness of either jaw tracking or CAO. Additionally, there were no apparent trends relating the effectiveness of jaw tracking to aspects of target geometry. In order to improve the ease at which patterns can be identified a larger sample size would be beneficial.

Though not tested in this study, in addition to target geometry a consideration should be made of the dosimetric impact of island blocking as a function of MLC width. Each of the patient plans included in this study were planned using a Varian Millennium 120 MLC. Therefore, each target, in each of the plans, was treated using 5 mm width MLCs. Theoretically, smaller MLC width will result in less island blocking and a comparison of this effect could be a topic of future study.

### 4.2 Quality assurance

The QA procedures in this study involved patient‐specific testing as recommended by the American College of Radiology (ACG) and American Society for Therapeutic Radiation Oncology (ASTRO) in AIP Conference Proceedings 2090.^25^

Though there was no statistically significant difference found for any sets of gamma analysis criteria and pass rates, there were still minor variations observed. Particularly, plans which included jaw tracking possessed slightly lower average passing rates and minimum passing rates, especially for the lower gamma analysis criteria sets. There were only small variations but as SIMT SRS requires very complex treatment plans,^25^, ^29^ even slight errors can have negative clinical impacts.^30^ The findings relating to jaw tracking of this research are supported by Wu et al.^15^ that compared identical plans with and without jaw tracking. Though all plans were deemed deliverable, it was found that the passing rate for plans with jaw tracking (96.9%) was slightly lower than with fixed jaws (99.2%) using gamma analysis criteria of 3%/3 mm.

When CAO was applied, there was no discernible difference between average or minimum pass rates indicating no change in plan deliverability with the addition of CAO. There is little to no literature regarding the deliverability of plans including CAO. A study by Ohira et al.^22^ suggested that due to a reduction in MUs and beam on time an improvement in safe dose delivery may be seen, however, this was not tested explicitly. As there was no change in MUs found in this research, this suggested improvement in deliverability is not expected.

## 5 CONCLUSION

This study analyzed the impact of two techniques upon treatment plan quality for plans delivered using SIMT SRS. In the 10 patients studied with 2–6 targets, contrary to expectations based upon previous literature, CAO had minimal effect upon dose‐level parameters used for clinical plan evaluation with Eclipse treatment planning optimizer. This is likely due to the use of the low‐dose optimization objective prompting the treatment planning system to compensate for island blocking, whereby the MLC leaves would close to treat targets individually in situations where island blocking would otherwise occur. Therefore, the findings from this research suggest that when using a low‐dose optimization objective the use of CAO may have limited benefit in treatment planning for VMAT‐based SIMT SRS for between 2 and 6 targets when using Eclipse treatment planning system.

Fitting with expectations, the application of jaw tracking showed statistically significant reductions in low‐dose level parameters, V10% and healthy brain mean dose. However, the reductions in absolute healthy brain volumes were small, and likely not clinically significant.

Comprehensive patient‐specific quality assurance procedures were carried out for all plans using a range of gamma analysis criteria sets. An apparent trend of lower average pass rates and minimum pass rates for jaw tracking plans, especially at the stricter gamma analysis criteria was observed. Further research into the deliverability of jaw tracking is still required as no statistical significance was found for this trend, possibly due to a limited sample size. For the patient cohort in this study, jaw tracking showed clinically minimal improvement in dose to healthy brain but also marginally worsened QA results. The minimal effect on heathy brain dose and worsened deliverability suggests caution toward the use of jaw tracking.

## CONFLICT OF INTEREST

No conflict of interest.

## AUTHOR CONTRIBUTIONS

Lauren Pudsey conducted project methods and wrote the manuscript with guidance and assistance of other authors. Dean Cutajar was a project supervisor. Alex Wallace, Anastasia Saba, and Laurel Schmidt created the original treatment plans used in this study. Andrej Bece and Catherine Clark provided clinical review and approval of the treatment plans used in this study. Yoshiya Yamada, Anatoly Rosenfeld, and Giordano Biasi provided guidance of the project as well as manuscript revision. Joel Poder was responsible for the overall idea of the project and assisted with development of methods and analysis of results. All authors were involved in review of the manuscript.

## Data Availability

The data that support the findings of this study are available from the corresponding author upon reasonable request.
